# Variability of FEV_1_ and Criterion for Acute Pulmonary Exacerbation

**DOI:** 10.3389/fped.2014.00114

**Published:** 2014-10-17

**Authors:** Bradlee A. Jenkins, Loyd Lee Glenn

**Affiliations:** ^1^Institute for Quantitative Biology, East Tennessee State University, Johnson City, TN, USA

**Keywords:** cystic fibrosis, functional expiratory volume, pulmonary, spirometry, prognosis, epidemiology, adolescents, *Pseudomonas aeruginosa*

Morgan et al. (1) concluded that cystic fibrosis (CF) in children and adolescents with a high baseline forced expiratory volume (FEV_1_) were less likely to have a therapeutic intervention or slower rate of FEV_1_ decline after a single acute decline in FEV_1_ of 10%. This conclusion is not well supported due to the arbitrary criteria used for defining a pulmonary exacerbation, as explained below.

First, only a single low FEV_1_ value defined an exacerbation. However, FEV_1_ measurements are notoriously variable from test-to-test; Taylor-Robinson et al. (2) showed that the baseline fluctuations have a wide range of about 60%. FEV_1_ tests are sensitive to time of day, health status, mood, tiredness, lack of sleep, medical instruction, nutritional status, acute comorbidities, and other factors Cystic Fibrosis Foundation (3). Given that a single FEV_1_ assessment was used, rather than the average of repeated measurements on different days, evidence that the assessment values were technically accurate.

Second, an exacerbation was defined as a single 10% decline in FEV_1_ in the above study without any explanation as to why 10% was chosen nor why a range of declines were not evaluated to determine the sensitivity and specificity of different criteria (by testing several criterion values such as 5, 15, 20, and 25%). A threshold of 10% is nearly within the noise level of the FEV_1_ measurements (2, 4). This leaves open the possibility that the findings in the study would not hold if a slightly greater (or smaller) value was used, such as 8 or 12%.

Furthermore, regarding the use of the cutoff of 10%, there is no agreement on the optimal cutoff for separating pulmonary exacerbations from the large natural technical variations in FEV_1_ from test-to-test, a variation that has been found to be higher in CF without evidence of concomitant changes in the severity of the condition (4, 5). National treatment guidelines (5) for acute pulmonary exacerbations in CF was based on the work of Fuchs et al. (6), a clinical trial of DNAase that only incidentally mentioned 10% as a criterion if 4 of 12 other signs or symptoms were present in a population of adults and children. In a more comprehensive study on children under six years of age, Rabin et al. (7) and Regelmann et al. (8) defined exacerbations operationally by whether or not pulmonologists decided to intensify treatment using antibiotics. These authors found the average FEV_1_ decline to be 20% in children under 6 years of age, these investigators proposed a refinement of the criterion to 15% or higher, rather than 10% suggested by Fuchs et al. (6), under the condition that a 15% decline would only be considered an exacerbation if two other clinical signs were concurrent, such as increased cough frequency, new crackles, or hemoptysis. Evidence on the best cutoff value is consequently nearly absent and remains to be determined. The most logical path follow would be to first test the full range of pulmonary decline criterion, perhaps from 0 to 30%, to determine the optimal criterion, and then to conduct a focused study on this value. The findings from such a range analysis could be of great value in helping further refine the criterion for an exacerbation based on FEV_1_ decline, rather than assuming a single value at this early stage.

The study has many prominent strength including well-defined hypotheses, very large sample size, analysis by age group, excellent statistical analysis, clearly presented findings, excellent flow of logic, and others. However, due to the shortcomings described above, we suggest further study of the above critical questions before the findings are implemented in practice.

## Conflict of Interest Statement

The authors declare that the research was conducted in the absence of any commercial or financial relationships that could be construed as a potential conflict of interest.

## References

[1] Morgan WJ Wagener JS Yegin A Pasta DJ Millar SJ Konstan MW Probability of treatment following acute decline in lung function in children with cystic fibrosis is related to baseline pulmonary function. J Pediatr (2013) 163:1152–7. 10.1016/j.jpeds.2013.05.013 23810128 PMC4064589

[2] Taylor-Robinson D Whitehead M Diderichsen F Olesen HV Pressler T Smyth RL Understanding the natural progression in % FEV_1_ decline in patients with cystic fibrosis: a longitudinal study. Thorax (2012) 67:860–6. 10.1136/thoraxjnl-2011-200953 22555277 PMC3446776

[3] Cystic Fibrosis Foundation. Cystic Fibrosis Foundation Patient Registry, 2010 Annual Data Report. Bethesda, MA: Cystic Fibrosis Foundation (2011).

[4] Cooper PJ Robertson CF Hudson IL Phelan PD. Variability of pulmonary function tests in cystic fibrosis. Pediatr Pulmonol (1990) 8:16–22. 10.1002/ppul.1950080107 2405342

[5] Flume PA Mogayzel PJ Jr Robinson KA Goss CH Rosenblatt RL Kuhn RJ Clinical practice guidelines for pulmonary therapies committee. Cystic fibrosis pulmonary guidelines: treatment of pulmonary exacerbations. Am J Respir Crit Care Med (2009) 180:802–8. 10.1164/rccm.200812-1845PP 19729669

[6] Fuchs HJ Borowitz DS Christiansen DH Morris EM Nash ML Ramsey BW Effect of aerosolized recombinant human Dnase on exacerbations of respiratory symptoms and on pulmonary function in patients with cystic fibrosis. The pulmozyme study group. N Engl J Med (1994) 331:637–42. 10.1056/NEJM199409083311003 7503821

[7] Rabin HR Butler SM Wohl ME Geller DE Colin AA Schidlow DV Epidemiologic study of cystic fibrosis. Pulmonary exacerbations in cystic fibrosis. Pediatr Pulmonol (2004) 37:400–6. 10.1002/ppul.20023 15095322

[8] Regelmann WE Schechter MS Wagener JS Morgan WJ Pasta DJ Elkin EP Investigators of the epidemiologic study of cystic fibrosis. Pulmonary exacerbations in cystic fibrosis: young children with characteristic signs and symptoms. Pediatr Pulmonol (2013) 48:649–57. 10.1002/ppul.22658 22949088 PMC4102401

